# Allergic sensitisation in elderly patients with rhinitis

**DOI:** 10.1186/1939-4551-8-S1-A27

**Published:** 2015-04-08

**Authors:** Andreea Ioana Popescu, Raluca Greblescu

**Affiliations:** 1 Pop De Basesti Medical Centre, Romania; 2 Medas Clinic, Romania

## Background

Available data suggest that allergen sensitisation declines with age, causing physicians to often disregard the allergic component in the pathogenesis of respiratory conditions in the elderly. Atopy is rarely considered in the clinical assessment of the geriatric rhinitis patients, and these patients are infrequently referred for allergy evaluation.

## Methods

The study included patients older than 65 years with rhinitis and an age- and gender-matched control group. Skin prick tests (SPT) with inhalant allergens (house dust mites, Alternaria, trees pollen, grass pollen, ragweed and mugwort pollen, animal dander) were performed on all the subjects. Detailed medical history was obtained and a questionnaire inquiring about the severity of symptoms, medication, family history of atopy was administered.

## Results

A total of 71 patients with rhinits / rhinoconjuctivitis (mean age 69.8 years) were recruited, 11 of them also had asthma. Twenty nine patients (40.8%) had at least one positive SPT result, compared to only 10 subjects (14%) in the control group. The most common allergic sensitisation was found to be to house dust mites in both groups. In the rhinitis group, 13 patients were found to be polysensitised. Symptom scores revealed that 44 patients (61.9%) assessed their nasal symptoms as severe.

## Conclusions

Despite the immune modifications occurring in the old age, the prevalence of allergic sensitisation in geriatric patients with rhinitis is substantial. If properly evaluated, these patients, who often present with severe symptoms, can benefit from preventive measures such as allergen avoidance and even specific immunotherapy.

